# Most unicompartmental knee replacement revisions could be avoided: a radiographic evaluation of revised Oxford knees in the National Joint Registry

**DOI:** 10.1007/s00167-020-05861-5

**Published:** 2020-02-10

**Authors:** James A. Kennedy, Jeya Palan, Stephen J. Mellon, Colin Esler, Chris A. F. Dodd, Hemant G. Pandit, David W. Murray

**Affiliations:** 1 Nuffield Department of Orthopaedics, Rheumatology and Musculoskeletal Sciences, University of Oxford, Oxford, UK; 2 Leeds Teaching Hospitals NHS Trust, Leeds, UK; 3 Division of Orthopaedic Surgery, Department of Health Sciences, University of Leicester, Leicester, UK; 4 Nuffield Orthopaedic Centre, Oxford University Hospitals NHS Foundation Trust, Oxford, UK; 5 Leeds Institute of Rheumatic and Musculoskeletal Medicine, University of Leeds, Leeds, UK

**Keywords:** Arthroplasty, Knee, Unicompartmental knee replacement, Revision, Registry

## Abstract

**Purpose:**

The purpose of this study was to understand why the revision rate of unicompartmental knee replacement (UKR) in the National Joint Registry (NJR) is so high. Using radiographs, the appropriateness of patient selection for primary surgery, surgical technique, and indications for revision were determined. In addition, the alignment of the radiographs was assessed.

**Methods:**

Oxford UKR registered with the NJR between 2006 and 2010 and subsequently revised were identified by the NJR. A blinded review was undertaken of pre-primary, post-primary, and pre-revision anteroposterior and lateral radiographs of a sample of 107 cases from multiple centres.

**Results:**

The recommended indications were satisfied in 70%, with 29% not demonstrating bone-on-bone arthritis. Major technical errors, likely leading to revision, were seen in 6%. Pre-revision radiographs were malaligned and, therefore, difficult to interpret in 53%. No reason for revision was seen in 67%. Reasons for revision included lateral compartment arthritis (10%), tibial loosening (7%), bearing dislocation (7%), infection (6%), femoral loosening (3%), and peri-prosthetic fracture (2%, one femoral, one tibial).

**Conclusions:**

Only 20% of the revised UKR were implanted for the recommended indications, using appropriate surgical technique and had a mechanical problem necessitating revision. One-third of primary surgeries were undertaken in patients with early arthritis, which is contraindicated. Two-thirds were presumably revised for unexplained pain, which is not advised as it tends not to help the pain. This study suggests that variable and inappropriate indications for primary and revision surgery are responsible for the high rates of revision seen in registries.

**Level of evidence:**

III, Therapeutic study.

## Introduction

There is discrepancy in reported revision rates for unicompartmental knee replacement (UKR). National joint registries all report UKR revision rates about three times higher than the most commonly used alternative, total knee replacement (TKR) [28–31]. The high revision rates have led to some authors calling for UKR to no longer be used. However, multiple large cohort studies that have used a mobile-bearing UKR as recommended have published revision rates substantially lower to that seen in registers, and equivalent to that seen in TKR [5, 21, 22, 24, 32, 39]. Furthermore, the recently published 5 year results of the Total Or Partial Knee Arthroplasty Trial (TOPKAT), a pragmatic randomised controlled trial (RCT) of over 500 patients, at 27 UK sites with 68 surgeons, has shown equivalent revision rates with TKR and UKR [1].

UKR is a less invasive operation than TKR and, as a result, major complications occur less frequently and mortality and morbidity is lower [2, 19, 26]. Better patient reported outcomes can be obtained with UKR [20], and patients recover quicker and can be discharged earlier so there are appreciable cost savings [3]. Up to 50% of knees requiring replacement satisfy the recommended indications for UKR [11, 13, 38], yet less than 10% are treated with UKR. If the causes for the high revision rates in registers could be identified and addressed then more patients could be treated with UKR with benefits to patients and the health service.

It is not clear why the revision rate of UKR in National Registers is so much higher than in many cohort studies and RCTs. Possible reasons may be that the indications for the primary or revision procedure, or the surgical technique are inappropriate. The aim of this study was to identify from the National Joint Registry of England, Wales, Northern Ireland and the Isle of Man (NJR) medial Oxford mobile-bearing UKR that had been revised and then based on radiographs to determine whether the (1) patient selection, (2) surgical technique, and (3) indications for revision were appropriate. In addition, (4) the alignment of the radiographs was assessed. The study hypothesis was that the reasons for the revisions were the same as those recorded by the NJR.

## Materials and methods

**Fig. 1:**
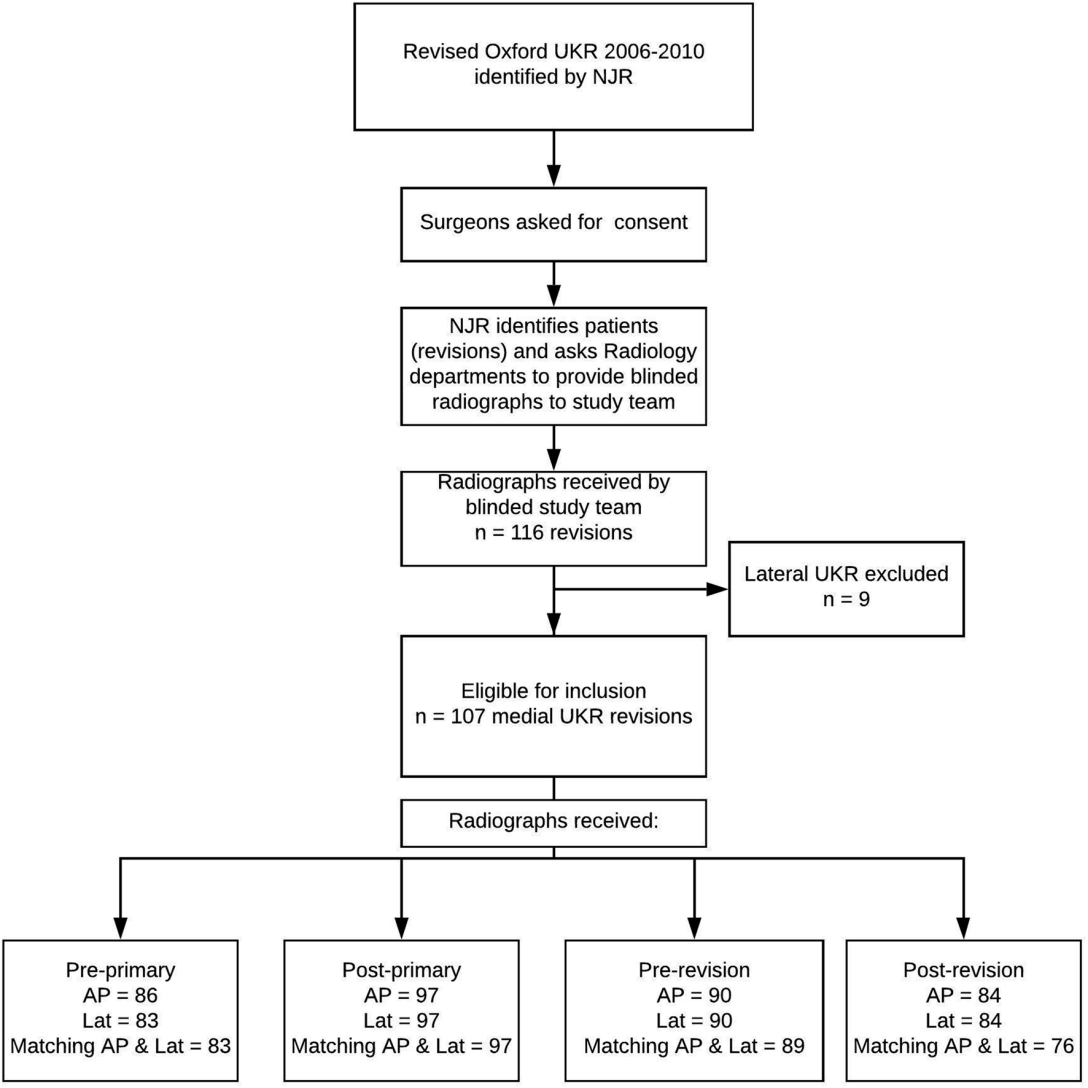
Study flow diagram. *UKR* unicompartmental knee replacement, *NJR* National Joint Registry, *AP* anteroposterior radiograph, *Lat* lateral radiograph

A nationwide, blinded retrospective cross-sectional service evaluation of revised UKR was designed in collaboration with the NJR (Fig. 1). Patients with a primary UKR with subsequent revision were identified by the NJR during January, 2013. The responsible surgeon was identified by the NJR and asked to consent for the study. Radiology departments of consenting surgeons were then asked to provide anteroposterior (AP) and lateral knee radiographs from immediately prior to the primary operation, immediately post primary operation, immediately prior to revision operation, and after revision operation. These radiographs were blinded and sent to the study team.

Medial Oxford mobile bearing UKR was chosen as the prosthesis for this study as it is the most commonly used UKR in the NJR [28], and can reliably be assessed radiographically [6]. It has evidence-based indications [10] and the decision whether to do a UKR or TKR is based on disease patho-anatomy identified with radiographs [11, 37]. Technical adequacy of the operation is also best assessed on aligned post-operative radiographs [15]. Revisions recorded by the NJR include any further operation requiring implant removal, addition or exchange, and should be performed if patients have unacceptable symptoms and an identified pathology. For mobile bearing UKR this is most commonly lateral osteoarthritis, aseptic loosening, dislocation of the bearing, or component overhang leading to soft tissue irritation. All of these and most other pathologies are reliably seen on plain radiographs. Revisions for infection are not common, but often show periarticular erosions, joint space narrowing or pathological radiolucencies. Furthermore, a spacer may be seen on the post revision radiograph.

All patients that received a primary medial Oxford UKR between 2006 and 2010, and had subsequent revision surgery as recorded by the NJR were eligible for inclusion. Inclusion criteria for analysis included the presence of AP and lateral knee radiographs. Multiple binary variables were constructed to denote the presence or absence of radiographic findings (Tables 1, 2, 3, 4). These include indications for surgery, operative adequacy, and indications for revision. There was one graded variable in the indication for primary operation section, which was the presence of bone-on-bone arthritis that was graded as: Bone-on-bone seen; Bone-on-bone not seen but possibly would have been seen on varus stress or Rosenberg radiographs; Bone-on-bone not seen and almost definitely not present. Lateral compartment osteoarthritis was determined via joint space narrowing with osteophytes ignored [11]. Deficiency of the anterior cruciate ligament (ACL) was assessed via the extent of posterior erosion of the medial tibial plateau as seen on a lateral radiograph [11, 17]. Operative adequacy was assessed using criteria similar to that used by Hurst et al. [15]. These are for example: femoral component varus/valgus < 10°, flexion 5° ± 10°, medial/lateral placement against the tibial spine; tibial component varus/valgus < 5°, postero-inferior tilt 7° ± 5°; depth of tibial saw cuts appropriate with minimal excess cement; no evidence of bearing impingement. Technical errors if present were graded as major if likely to cause implant failure (e.g., extreme implant malposition), or minor if they were unlikely to cause implant failure.

A custom written graphical user interface was constructed using Matlab (MATLAB Release 2017b, MathWorks, Inc) to facilitate the viewing of large numbers of radiographs. Radiographs were reviewed by two authors (*JK, DM*) and consensus decision reached regarding the presence or absence of radiographic findings. Radiographs from 30 knees (28%) were retested after two months. Agreement was substantial to almost perfect (unweighted Cohen’s Kappa statistic for: indications 0.90; presence of technical errors 0.71; revision indication 0.94; and radiograph alignment 0.68).

### Ethics, funding and conflict of interest

Ethical approval was sought but deemed unnecessary by the local research ethics council, as all investigations were part of routine care. This work was supported by the Orthopaedics Trust (Gwen Fish Fund). The author or one or more of the authors have received or will receive benefits for personal or professional use from a commercial party related directly or indirectly to the subject of this article. In addition, benefits have been or will be directed to a research fund, foundation, educational institution, or other non-profit organisation with which one or more of the authors are associated.

### Statistical methods

Counts were used to tally the number of findings. Percentages were calculated against the number of eligible radiographs. As an exploratory descriptive study, a sample size calculation was not performed. Unweighted Cohen’s Kappa statistics were calculated for retest reliability, and considered substantial if the statistic was 0.61–0.80, and almost perfect if it was 0.81–1.00.

## Results

Radiographs were received for 107 medial UKR. Matching AP and lateral pre-primary radiographs were received for 78%, post-primary for 91%, pre-revision for 83% and post-revision films for 71% (Fig. 1).

### Indications for primary UKR

**Fig. 2:**
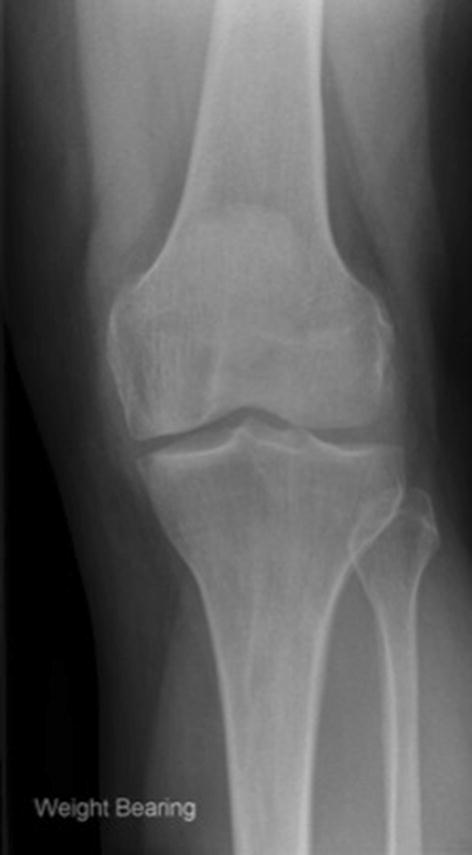
A preoperative weight-bearing anteroposterior radiograph demonstrating preserved medial joint space. This represents partial thickness cartilage loss and is a contraindication to unicompartmental knee replacement (UKR); UKR performed in these patients have a higher incidence of reoperation, revision and persistent post-operative pain

The majority of patients met the indications for surgery (58/83 with preoperative imaging, 70%; Table 1). Twenty-four (29%) did not demonstrate bone-on-bone osteoarthritis. In 16 (19%) the joint space was normal or nearly normal so it was virtually impossible for there to be bone-on-bone (Fig. 2). There were 2 (2%) cases of spontaneous osteonecrosis of the knee, both involving the medial femoral condyle.

**Table 1:** Indications for primary surgery (adequate radiographs *n* = 83)

Indications satisfied	58 (70%)
Reason indications not satisfied^a^	
Bone-on-bone not seen but might be seen on stress or Rosenberg X-rays	8 (10%)
Bone-on-bone definitely not present	16 (19%)
Lateral OA	2 (2%)
ACL deficiency	1 (1%)
Previous HTO	2 (2%)
Other contraindication	2 (2%)

*OA* osteoarthritis, *ACL* anterior cruciate ligament, *HTO* high tibial osteotomy

^a^Knees can have more than one contraindication

### Technical outcome of primary UKR

**Fig. 3:**
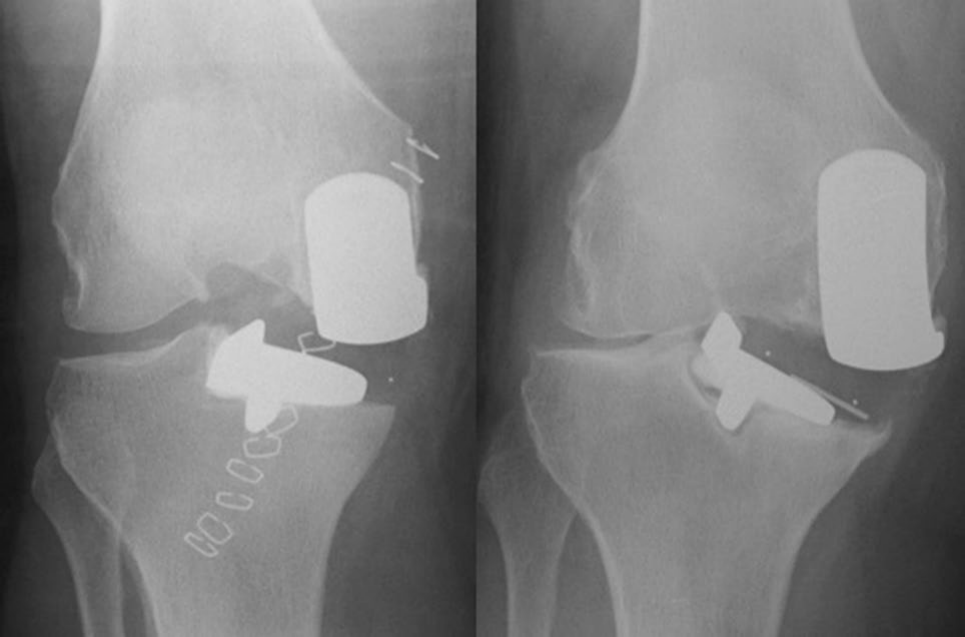
A post-primary and pre-revision radiograph of a poorly positioned tibial component leading to tibial loosening

**Fig. 4:**
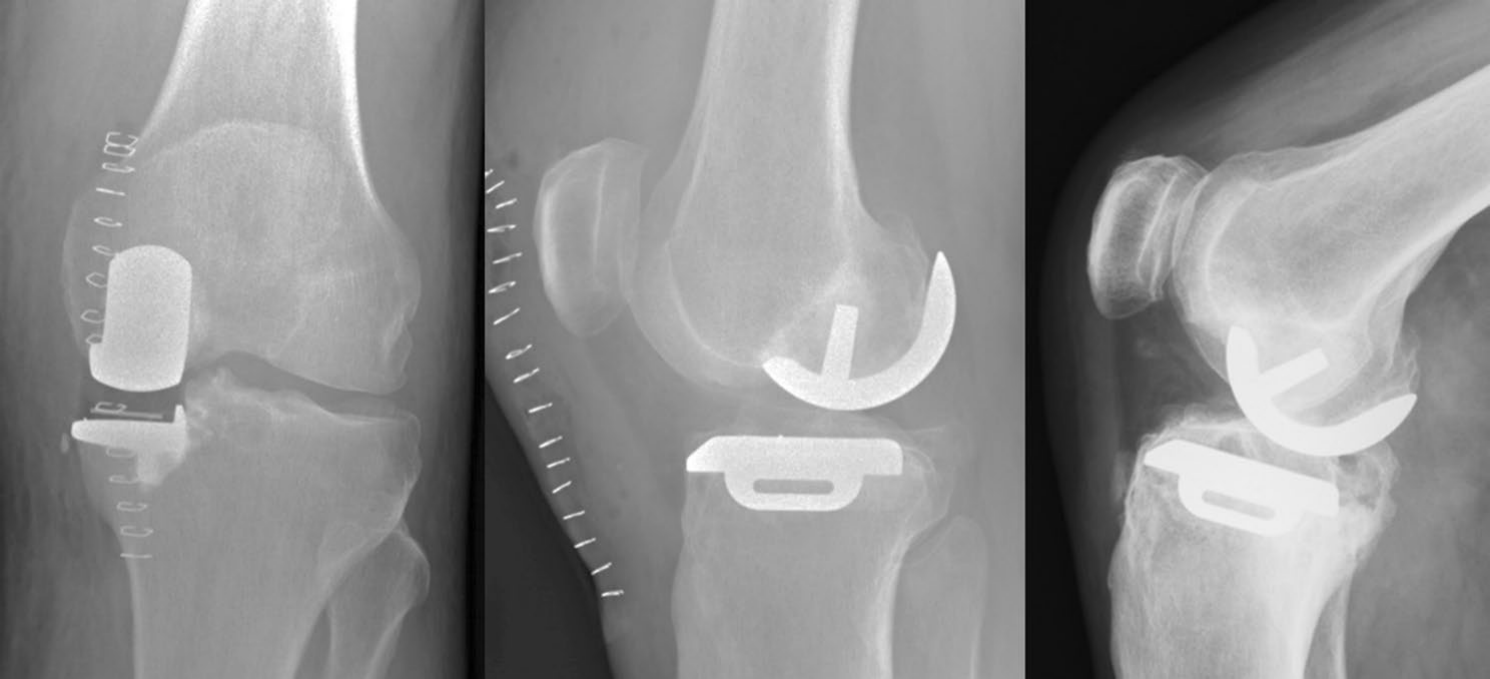
A medial tibial cut leading to tibial component undersizing and posterior underhang. The posterior tibial tray subsequently subsided into the  cancellous bone

**Fig. 5:**
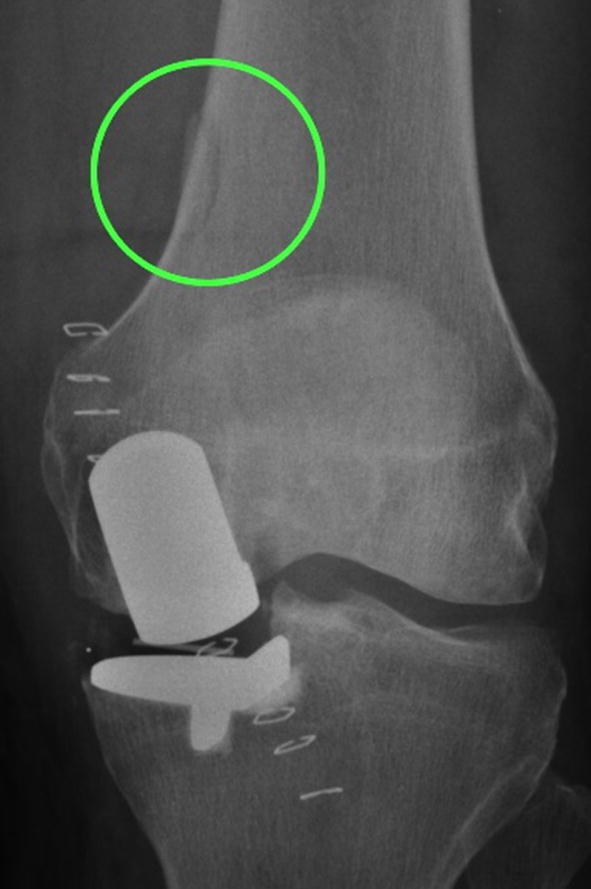
A post-primary anteroposterior radiograph demonstrating a malaligned femoral component, which is likely due to a malpositioned intramedullary guide rod that has pierced the femoral cortex (circled)

Post-primary or pre-revision radiographs were available for 106 patients, allowing assessment for technical factors. Many surgical errors were noted (62%; Table 2). Major errors were seen in 6 (6%), and minor errors in 58 (56%). All major errors were related to the tibial cut/component related (Figs. 3 and 4). Minor errors were most frequently tibial cut errors (i.e. medial, deep and/or varus cuts). Undersized tibial components were recorded in nine cases, and in eight of these were associated with medial cuts. Additional errors included two cases with valgus femoral components which demonstrated exit points for the femoral intramedullary guide rod (Fig. 5), one case with retained posterior osteophyte, and five cases appeared not to have had bone removed anterior to the femoral component which would lead to impingement.

**Table 2:** Technical errors identified (*n* = 104)

Major (*n* = 6, 6%)	
Tibial cut errors	5 (5%)
Tibial component undersize	2 (2%)
Minor (*n* = 58, 56%)	
Tibial cut errors	40 (38%)
Femoral cut errors	6 (6%)
Cementation errors	11 (11%)
Component malsizing	7 (7%)
Miscellaneous^a^	7 (7%)
None (*n* = 40, 38%)	

^a^Includes failure to remove anterior bone from femoral cut, failure to remove posterior osteophytes, and possible bearing overstuffing or medial collateral ligament damage

### Indication for revision surgery

**Fig. 6:**
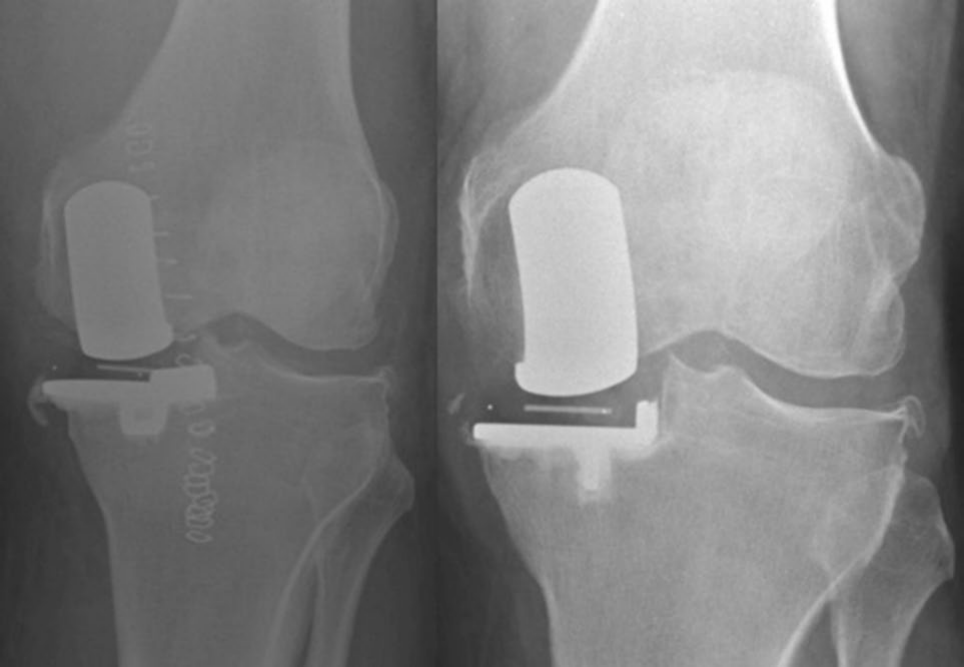
Radiograph on the left demonstrating what appears to be significant medial overhang and malalignment of the tibial component. A subsequent aligned radiograph of the same knee demonstrating a perfectly aligned tibial component. Note the presence of excess cement in and around the joint

In 60 (67%; Table 3) pre-revision radiographs, a reason for revision was not identified. Common causes for revision were lateral compartment arthritis (10%), aseptic loosening (10%), and dislocation (7%). Infection was the cause for revision in 6% as spacers were seen on the post-revision radiographs. Other causes included peri-prosthetic fracture (2%, one femoral, one tibial) and gross component mal-alignment (2%). However, some cases had malaligned radiographs that made the implant look malaligned and malpositioned, but looked well aligned and positioned on aligned films (Fig. 6).

**Table 3:** Identified reasons for revision (*n* = 89)

None identified	60 (67%)
Disease progression	9 (10%)
Tibial loosening	6 (7%)
Dislocated bearing	6 (7%)
Infection	5 (6%)
Femoral loosening	3 (3%)
Malalignment	2 (2%)
Periprosthetic fracture	2 (2%)
Cement in joint	1 (1%)

### Quality of radiographs

Radiographic malalignment in immediate post primary surgery films was noted in 48 (49%, Table 4) AP radiographs, and 3 (3%) lateral radiographs. In the pre-revision surgery films, AP malalignment was noted in 48 AP radiographs (53%), and 3 lateral radiographs (3%).

**Table 4:** Radiograph malalignment (*n* = 97 postop, *n* = 90 pre-revision)

Post op AP	48 (49%)
Post op lateral	3 (3%)
Pre revision AP	48 (53%)
Pre revision lateral	3 (3%)

*AP* anteroposterior

## Discussion

This study has identified the main reasons why the revision rate of UKR is high in the NJR, and much higher than in large cohort studies and RCTs [1, 5, 21, 22, 32, 39]. These include revisions performed without radiographic evidence of joint failure (67% of cases), inappropriate patient selection for primary surgery (30% of cases), and major technical errors with the operation (6% of cases). Furthermore, there were many inadequately aligned radiographs making assessment difficult. Taken together only 20% of the revisions had appropriate indications for the primary procedure, appropriate surgical technique and an identifiable reason for revision. Therefore, potentially 80% were avoidable.

This study has limitations. Although this study of 107 revisions is the largest radiographic study of revised knee replacements, it represents a sample of revised UKR in the NJR. There is, however, no reason to believe that the radiographs from patients in the sample were any different from those not in the sample. In particular, whether radiographs were received depended on the response of the local ethics and radiology departments and was independent of surgeon and patient, ensuring that the sample was representative and unbiased. Evidence for this is provided by the close approximation in the rates of clear pathologies leading to revision in this study and in the NJR (Table 5). This was particularly important for infection, as those with one-stage revisions might not have been identified. Another limitation was that no data were provided by the NJR about the patients and patient reported outcomes were not available. As a result the patient’s status before revision, or if they improved following revision, was not known. Finally, the decision to revise is not based on radiographs alone. However, other imaging modalities that are occasionally used, such as bone scan or MRI, tend not to help in this situation or can be misleading. In addition some patients will have had arthroscopy, which occasionally identifies pathology, but this is rare.

**Table 5:** Study vs NJR. Failure mode as percentage of all revisions

	Study	NJR UKR	Difference
Pain		25%	N/A
Dislocation/subluxation	7%	6%	1%
Infection	6%	5%	1%
Aseptic loosening	10%	28%	18%
Lysis	0	4%	4%
Peri-prosthetic fracture	2%	2%	0%
Implant fracture	0	0.3%	0%
Implant wear	0	8%	8%
Instability		8%	N/A
Mal-alignment	2%	6%	4%
Other	17%	36%	19%
Stiffness		2%	N/A

The indications for mobile-bearing UKR are evidence based, well defined and are based on patho-anatomy. The primary indication is anteromedial osteoarthritis, with bone-on-bone arthritis medially, full thickness lateral cartilage, and functionally intact ligaments [11, 37]. In 29% the pre-operative radiographs did not show bone-on-bone arthritis. In some of these, which had marked joint space narrowing, there may have been true bone-on-bone arthritis, which would have been apparent had a Rosenberg or varus stress radiograph been available. However, in 19% there was virtually full thickness medial joint space and there could not have been bone-on-bone arthritis (Fig. 2). The results of UKR used in this situation are unpredictable and persistent pain, reoperations and revisions are common [9, 27]: In one study, the reoperation/revision rate was about 60% when the pre-operative radiographs showed near normal joint space [27]. In only 3% of cases was the disease more severe than recommended (2% with lateral joint space narrowing and 1% with radiographic evidence of a non-functional ACL). The recommended indications are satisfied in up to 50% of knee replacements [11, 13, 38], yet the NJR shows that UKR is used in only 10% of cases [28]. This study, therefore, suggests that many surgeons are implanting UKR in patients with early disease, without bone-on-bone arthritis, and are not adhering to the recommended indications. These surgeons probably feel that UKR should be used in patients whose disease is not severe enough for TKR. This is not recommended and results in surgeons doing small numbers of UKR and having poor results. UKR should be considered to be an alternative treatment option to TKR for patients with bone-on-bone arthritis. To achieve the best results with the mobile bearing UKR, surgeons should adhere to the recommended indications so they use UKR for at least 20% and ideally about 50% of their primary knee replacements [12].

Although surgical errors were noted in 62% of the operations, only 6% were considered to be major, meaning they would cause symptoms and require a revision. The remainder were considered minor implying they probably would not compromise the outcome. However, if for some unrelated reason, the patients had symptoms, surgeons unfamiliar with the device might do a revision believing a minor surgical error was responsible for the symptoms. The majority of errors were with tibial cut height and tibial component orientation, which commonly occurred with the instrumentation (Phase 3) used at that time. New instrumentation (Microplasty) is now used and includes a stylus with slotted saw guides to control cut height and orientation, and a system that links the femoral drill guide to an intramedullary rod to improve femoral component orientation [18, 23, 36]. Cementation errors were also noted frequently, and the introduction of cementless components should prevent these. Undersized tibial components are at risk of subsidence, and the majority of these were associated with a vertical cut that was too far medial (Fig. 4). The vertical cut should be just medial to the apex of the medial spine.

It is generally accepted that TKR should not be revised for unexplained pain, as under these circumstances revision surgery is unlikely to be of benefit [16, 25, 35]. The same recommendation applies to UKR although the data supporting this is very limited and further study is needed [6, 33]. Two out of three patients (67%) did not demonstrate a radiographic reason for revision. The majority of these will have had a revision for unexplained pain, and they are unlikely to have benefited from this. This is one of the main reasons why the revision rate of UKR is higher than TKR in National Registries, and it probably also explains why the re-revision of UKR rate is high. The biggest problem occurs in patients who had the primary UKR for pain without bone-on-bone-arthritis, as it is likely that the primary surgery will not help, so they will have a revision, which will probably also not help, and so they will undergo a re-revision. An important advantage of UKR compared to TKR is that it is much easier to revise as a revision is usually a simple conversion to a primary TKR [34]. As a result many surgeons revise a UKR for unexplained pain whereas most surgeons avoid revising a TKR for unexplained pain. There is evidence from the New Zealand Joint Registry that this results in a different threshold for revision: Patients who have a bad outcome score following TKR have about a 10% chance of being revised whereas UKR with an equally bad score have a 60% chance of being revised [7]. As a result, even though UKR have less bad results than TKR they have a higher revision rate.

For most of the failure modes the proportion of revisions in this study were similar to that reported by the registry, suggesting the radiographic review is reliable (Table 5). For example the revision rate due to dislocation (7%), infection (6%), and peri-prosthetic fracture (2%) are virtually identical. However, for some indications they were very different, which disproves the study hypothesis that the revision rates recorded by the NJR would be the same as found in this study. Pain was reported as being the cause of revision by the NJR in 25% of cases, whereas this study suggests the incidence of revision for unexplained pain is much higher. A possible explanation for this is that surgeons know that revising a knee replacement for unexplained pain is not recommended so tend not to record this. Aseptic loosening was present in 11% in this study which is much lower than reported in the NJR (28%). It may be that surgeons over report the incidence of tibial loosening, partly because a secure cemented tibial component is easy to dislodge if hit with a hammer and partly because of misinterpretation of radiolucent lines. Narrow radiolucent lines, otherwise known as physiological radiolucencies, are commonly present beneath the tibial component and are not indicative of loosening or a source of pain [8]. However, surgeons not familiar with mobile bearing UKR may feel they are indicative of loosening. The other main cause of revision in this study was lateral OA (10%), which cannot be compared to the NJR as this data is not recorded by the NJR.

**Fig. 7:**
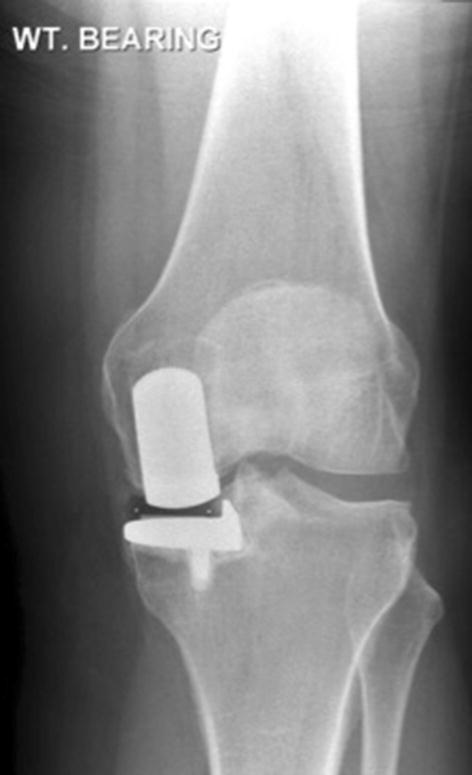
A malaligned AP film with a possible radiolucency. The only way to determine if there is a radiolucency and if it is pathological or physiological is with an aligned radiograph

There was variability in the quality of post-operative radiographs. It is recommended that anteroposterior radiographs are aligned to the tibial component, and lateral radiographs aligned to the femoral component [6]. On malaligned radiographs correctly positioned components may appear mal-positioned or malaligned (Fig. 6), and this may incorrectly be interpreted as a cause of pain. A problem peculiar to the Oxford Knee is that the posterior part of the tibial component is wider than the tibia to support the bearing in high flexion. Therefore, with malaligned radiographs postero-medial tibial overhang is often seen but there is no evidence that this causes pain and it not a justification for a revision (Fig. 6). In contrast with well aligned radiographs if tibial overhang is seen this means there is medial overhang in the region of the MCL, which, if extensive, can cause pain and be a justification for revision [4]. The only way to be certain if there is component loosening is if there is component migration. To assess migration requires two sequential radiographs to be taken in an identical fashion, which requires aligned radiographs. The assessment of the bone implant interface and whether a radiolucency is physiological or pathological can also only be done with an aligned radiograph. With an oblique view the interface is obscured (Fig. 7). It is, therefore, important that radiographers are taught how to take aligned radiographs [14].

This was logistically a difficult study to conduct, which is perhaps not surprising as it was the first nationwide multicentre radiographic study based on the NJR. However, having identified many pitfalls, a similar study would be much easier in the future. The advice by the Local Research Ethics Committee was that the study was considered to be a service evaluation (audit) and, therefore, did not require ethical approval. This resulted in much resistance from local ethics committees in the collaborating centres. A recommendation for future studies would, therefore, be to obtain a formal letter from the outset explaining why ethical approval is not required. When surgeons were contacted, virtually all (95%) gave approval. Thereafter the NJR requested radiographs from the consenting surgeon’s radiology department. This was done by a single letter to the “head of radiology” and no reminders or follow up was undertaken. For confidentiality reasons the study centre was not provided with patient details, so further requests for the radiographs were not possible. Thus another recommendation would be to have a mechanism by which the study centre can liaise with local radiology departments. Furthermore, obtaining patient information from the registry is recommended so that it can be matched with the radiographs and inform analyses.

## Conclusions

This study has identified the main reasons for the high rate of revision of UKR seen in national registries: in two thirds of cases the revision was done for unexplained pain, with no mechanical problem. Although further study is needed, the limited available evidence that exists suggests that revision in this situation does not help and, therefore, should be avoided. In one third of cases the primary procedure was done inappropriately in early arthritis without bone-on-bone. Although minor surgical errors were common, major errors leading to revision occurred rarely. With improved education around indication for primary and revision surgery and improved instrumentation it is likely that not only would the revision rate decrease but the number of UKR implanted would increase. This in turn would result in improved outcomes for patients needing knee replacement.
